# Fresh Insights Into *SLC25A26*: Potential New Therapeutic Target for Cancers: A Review

**DOI:** 10.3389/or.2024.1379323

**Published:** 2024-04-30

**Authors:** Yangheng Xu, Zhisheng Hong, Sheng Yu, Ronghan Huang, Kunqi Li, Ming Li, Sisi Xie, Lvyun Zhu

**Affiliations:** ^1^ Science and Engineering, National University of Defense Technology, Changsha, China; ^2^ The Second School of Clinical Medicine, Southern Medical University, Guangzhou, China; ^3^ Department of Biology and Chemistry, College of Sciences, National University of Defense Technology, Changsha, China

**Keywords:** *SLC25A26*, S-adenosylmethionine carrier protein, cancer, gene expression regulation, methylation

## Abstract

*SLC25A26* is the only known human mitochondrial S-adenosylmethionine carrier encoding gene. Recent studies have shown that *SLC25A26* is abnormally expressed in some cancers, such as cervical cancer, low-grade glioma, non-small cell lung cancer, and liver cancer, which suggests *SLC25A26* can affect the occurrence and development of some cancers. This article in brief briefly reviewed mitochondrial S-adenosylmethionine carrier in different species and its encoding gene, focused on the association of *SLC25A26* aberrant expression and some cancers as well as potential mechanisms, summarized its potential for cancer prognosis, and characteristics of mitochondrial diseases caused by *SLC25A26* mutation. Finally, we provide a brief expectation that needs to be further investigated. We speculate that *SLC25A26* will be a potential new therapeutic target for some cancers.

## Introduction

**FIGURE 1:**
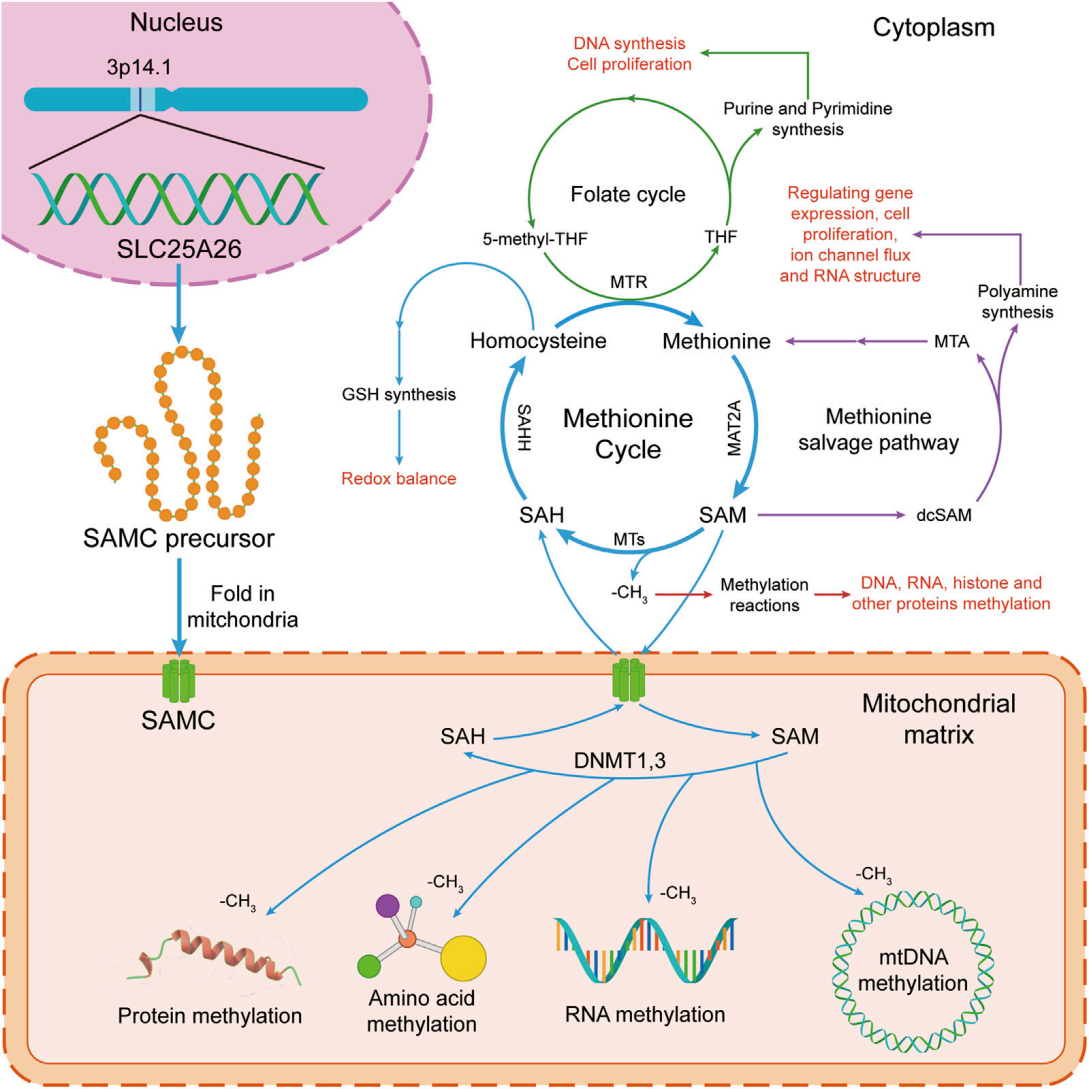
The S-adenosylmethionine (SAM) transport and function in human cells. In the cytoplasm, SAM takes part in the methionine cycle and polyamine synthesis. In mitochondria, SAM donates a methyl group for methylation reactions. The methionine cycle is interconnected with the transsulfuration pathway, the folate cycle, the methionine salvage pathway, and polyamine synthesis, all of which maintain important cellular functions. Firstly, SAM is converted by methionine via methionine adenosyltransferase 2A (MAT2A). Secondly, after donating a methyl group for methylation reactions, SAM is converted into S-adenosylhomocysteine (SAH) by methyltransferases (MTs). Then, SAH is then hydrolyzed by S-adenosyl-L-homocysteine hydrolase (SAHH) to generate homocysteine. Finally, homocysteine becomes methionine by receiving a methyl group from the folate cycle, which is mediated by 5-methyltetrahydrofolate: homocysteine methyltransferase (MTR). At the same time, SAM is transported to mitochondria for methylation reactions of mitochondrial DNA, RNA, protein and amino acid by mitochondrial S-adenosylmethionine carrier (mSAMC) which is encoded by *SLC25A26* nuclear gene on chromosome 3p14.1. GSH, glutathione; MTR, 5-methyltetrahydrofolate: homocysteine methyltransferase; SAHH, S-adenosyl-L-homocysteine hydrolase; MTs, methyltransferases; MAT2A, methionine adenosyltransferase 2A; SAM, S-adenosylmethionine; SAMC, mitochondrial S-adenosylmethionine carrier; SAH, S-adenosylhomocysteine. THF, tetrahydrofolate; 5-methyl-THF, 5-methyltetrahydrofolate.

The mitochondrial S-adenosylmethionine carrier (mSAMC), a member of the mitochondrial carrier family (MCF), widely exists in all eukaryotes and is located in the mitochondrial inner membrane [1–3]. The mSAMC catalyzes the import of S-adenosylmethionine (SAM) from the cytosol into the mitochondria and the export of S-adenosylhomocysteine (SAH) from the mitochondria into the cytosol [4–11]. In the cytoplasm, SAM participates in the methionine cycle and polyamine biosynthesis (Figure 1) [12–18]. In the mitochondria, SAM provides the methyl for methylation of DNA, RNA, proteins, and amino acids (Figure 1). Therefore, SAM has a great influence on sustained epigenetic modifications and has been implicated in several diseases and potential pathogenesis of cancer development [19]. For example, high or extremely high activity of methionine cycle metabolism was observed in cancer cells, especially tumor-initiating cells or tumor stem cells. Moreover, the proliferation of tumor cells is significantly inhibited by drug intervention of methionine cycle metabolism [20]. Schober et al. reported that mSAMC is the only carrier for transporting SAM from cytoplasm to mitochondria, and mitochondrial SAM levels in flies, mice, and humans are directly dependent on cytosolic SAM generated in the methionine cycle. Therefore, *SLC25A26* abnormal expression or mSAMC dysfunction can be associated with multiple disease states, including cancer, nutrient deficiencies, and cardiovascular diseases [21]. The mitochondrial carrier family in humans, called solute carrier family 25 (SLC25), has been identified for 53 members which are widely distributed in eukaryotes with similar structure and are mainly embedded in the inner membrane of mitochondria. They are involved in the transmembrane transport of various substrates including amino acids and their derivatives, cofactors, inorganic ions, nucleotides, and so on, which is further associated with mitochondrial metabolism and cell function [3, 22, 23]. Human mSAMC was encoded by solute carrier family 25 member 26 (*SLC25A26*) gene [9]. Growing studies showed that aberrant expression of *SLC25A26* may be involved in the occurrence and development of some cancers [24–32].

Therefore, we will briefly review mSAMC in different species and its encoding gene, focusing on the association of *SLC25A26* gene aberrant expression and some cancers as well as potential mechanisms, and characteristics of mitochondrial diseases caused by *SLC25A26* mutation. Finally, we provide a brief expectation that needs to be further investigated.

## Mitochondrial S-Adenosylmethionine Carrier and Encoding Gene in Different Species

To date, mSAMC was identified in *saccharomyces cerevisiae* in 2003, human in 2004 and Arabidopsis in 2006 [5, 9, 10]. In *Saccharomyces cerevisiae*, it was named Sam5p (Pet8p is used now) and encoded by *PET8* gene [5, 33–37]. In human, it was named SAMC and encoded by *SLC25A26* gene which is located on chromosome 3p14.1 as an autosomal recessive hereditary double allele gene with a highly conserved exon similar to the other mitochondrial carrier genes [38]. In Arabidopsis, it has two isoforms, named SAMC1 and SAMC2 which were encoded by *At4g39460 and At1g34065* gene, respectively. Sam5p (Pet8p), SAMC, SAMC1, and SAMC2 have the characteristics of the MCF and are located at mitochondria [3, 39]. However, later research proved that SAMC1 also exists on the chloroplast envelope membrane [24, 40–42]. SAMC1, SAMC2, and Sam5p (Pet8p) catalyze countertransport between SAM and mitochondrial SAH (mainly), S-adenosylcysteine (SAC), or adenosylornithine [5]. SAMC catalyzes countertransport between SAM and mitochondrial SAH [9]. SAMC1 and SAMC2 catalyze countertransport between SAM and mitochondrial SAH (mainly) or SAC [10]. Table 1 showed the differences and function of the mitochondrial S-adenosylmethionine carrier in yeast, human, and Arabidopsis, respectively.

**TABLE 1:** Comparison of mSAMC in yeast, human and Arabidopsis.

	In yeast	In human	In Arabidopsis
Identified time	2003 [5]	2004 [9]	2006 [10]
Name	Sam5p (PET8)	SAMC	SAMC1, SAMC2
Encoding gene	*YNL003c*	*SLC25A26*	*AT4G39460, AT1G34065*
Amino acid length	284	274	325, 321
Structure	Six transmembrane domains are arranged in chimney-shape	Six transmembrane domains are arranged in chimney-shape	Six transmembrane domains are arranged in chimney shape, with a longer polypeptide chain blocking the channel
Function	Catalyzes countertransport between SAM and mitochondrial SAH (mainly), SAC or adenosyl ornithine	Catalyzes countertransport between SAM and mitochondrial SAH	Catalyzes countertransport between SAM and mitochondrial SAH (mainly) or SAC
Location	Inner mitochondrial membrane	Inner mitochondrial membrane	Inner mitochondrial membrane and chloroplast envelope membrane

Additionally, the 2004 *in vitro* study on human recombinant SAMC identified several potent inhibitors [9]. These include pyridoxal 5′-phosphate, p-hydroxymercuribenzoate, mersalyl, and mercuric chloride, which are general inhibitors of mitochondrial carriers, and tannic acid and Bromocresol Purple, which specifically inhibit glutamate carriers. Notably, inhibitors characteristic of other mitochondrial carriers showed minimal impact on the activity of recombinant SAMC.

Regarding the regulation of the *SLC25A26* gene, FOXD3 has been identified as a repressor in the CaSki cell line [25], while CTB enhances senescence in HCC cells by inducing the upregulation of *SLC25A26* expression, potentially influencing its activation [28].

## *SLC25A26* Aberrant Expression May Regulate the Occurrence and Development of Some Cancers

In some cancers, *SLC25A26* expression is aberrant (Table 2). Patients with invasive cervical cancer have recurrent chromosome 3p12-p14 loss. In 2013, Lando et al. reported that 8 genes including *SLC25A26* were highly downregulated in invasive cervical cancer patients with recurrent chromosome 3p12-p14 loss, which may be associated with cancer invasiveness [43]. In cervical cancer line CaSki and HeLa cells, *SLC25A26* gene is also downregulated [26]. A large-scale genome-wide association study of low-grade glioma revealed 2 causal single nucleotide polymorphisms at rs11706832 site. One is rs11706832 reference allele: A, which may generate a binding site for a transcriptional repressor named LEF1 to inhibit *SLC25A26* expression. The other is rs11706832 alternative allele: C, a risk alternative allele, which promotes *SLC25A26* expression [27]. In human liver cancer tissues, *SLC25A26* expression was low compared to adjacent tissues. A novel copper complex [Cu(ttpy-tpp)Br_2_]Br (Referred to as CTB) exerted an anti-hepatocellular carcinoma effect by up-regulating *SLC25A26* expression level in mice [28]. In non-small cell lung cancer, expression of *SLC25A26* and the 10-year survival rate of the patients were negatively correlated [31]. In most colorectal cancer patients, *SLC25A26* gene was highly expressed. Moreover, in a subcutaneous transplanted tumor model with MC38 cells, a mouse colon cancer cell lines, knockdown of *SLC25A26* caused tumor volume to begin to decrease by day 9 and completely disappear by day 30, whereas control tumor volume continued to increase [32]. The expression of *SLC25A26* in different cancers is shown in Table 2. These studies suggest that aberrant expression of *SLC25A26* may be associated with the occurrence and development of some cancers.

**TABLE 2:** Altered *SLC25A26* expression and its effects on some cancers.

Types of cancer	*SLC25A26* expression level	Effect
Invasive squamous cell carcinoma of the uterine cervix [43]	Highly down-regulated due to recurrent chromosome 3p12-p14 loss	May be associated with increased invasiveness, treatment resistance, and poor prognosis
Cervical cancer line CaSki and HeLa cells [26]	Down-regulated due to promoter hypermethylation	NG (Not Given)
	Manually up-regulated	● Hypermethylated mtDNA (especially D-loop control region)
		● Impaired mitochondrial oxidative phosphorylation
		● Increased reactive oxygen species
		● Reprogrammed methionine cycle leading to decreased GSH
		● Inhibited cell growth, promoted cell apoptosis, and arrested cell cycle (S phase)
		● Increased cisplatin chemosensitivity
Low-cells grade gliomas [27]	Suppressed due to reference allele rs11706832-A might bind with LEF1	NG
	Increased due to risk allele rs11706832-C	NG
Hepatocellular carcinoma [28]	Down-regulated	High expression of proliferation index Ki67
		Low expression of senescence markers p16, p21, HMGA1
	Up-regulated due to CTB treatment	Mitochondrial dysfunction (impaired ATP generation)
		Impaired methionine cycle
		Cell senescence:
		● Increased p16, p21, and HMGA1 expression
		● Significantly increased G1 phase ratio and reduced S phase ratio
		Epigenetic Changes:
		● Reduced cytoplasmic SAM levels lead to TERT hypomethylation, which may contribute to the suppression of TERT expression and the senescence process
		● Increased mitochondrial SAM levels lead to hypermethylation of mtDNA, such as D-loop and mtCOX, which affects the expression of mitochondrial respiratory complex subunits
Non-small cell lung cancer [31]	Up-regulated	Increased risk-score and reduced 10-year survival rate of patients
Most colorectal cancer [32]	Up-regulated	Positively correlated with immune checkpoint gene LAG3, CD244, CD274, KIR3DL1, PDCD1, IDO1, VTCN1 expression
Mouse colon cancer MC38 cell lines [32]	Manually knocked out	Transplanted tumours in mice almost disappeared

## Potential Mechanisms for *SLC25A26*-Regulating the Occurrence and Development of Cancers

**FIGURE 2:**
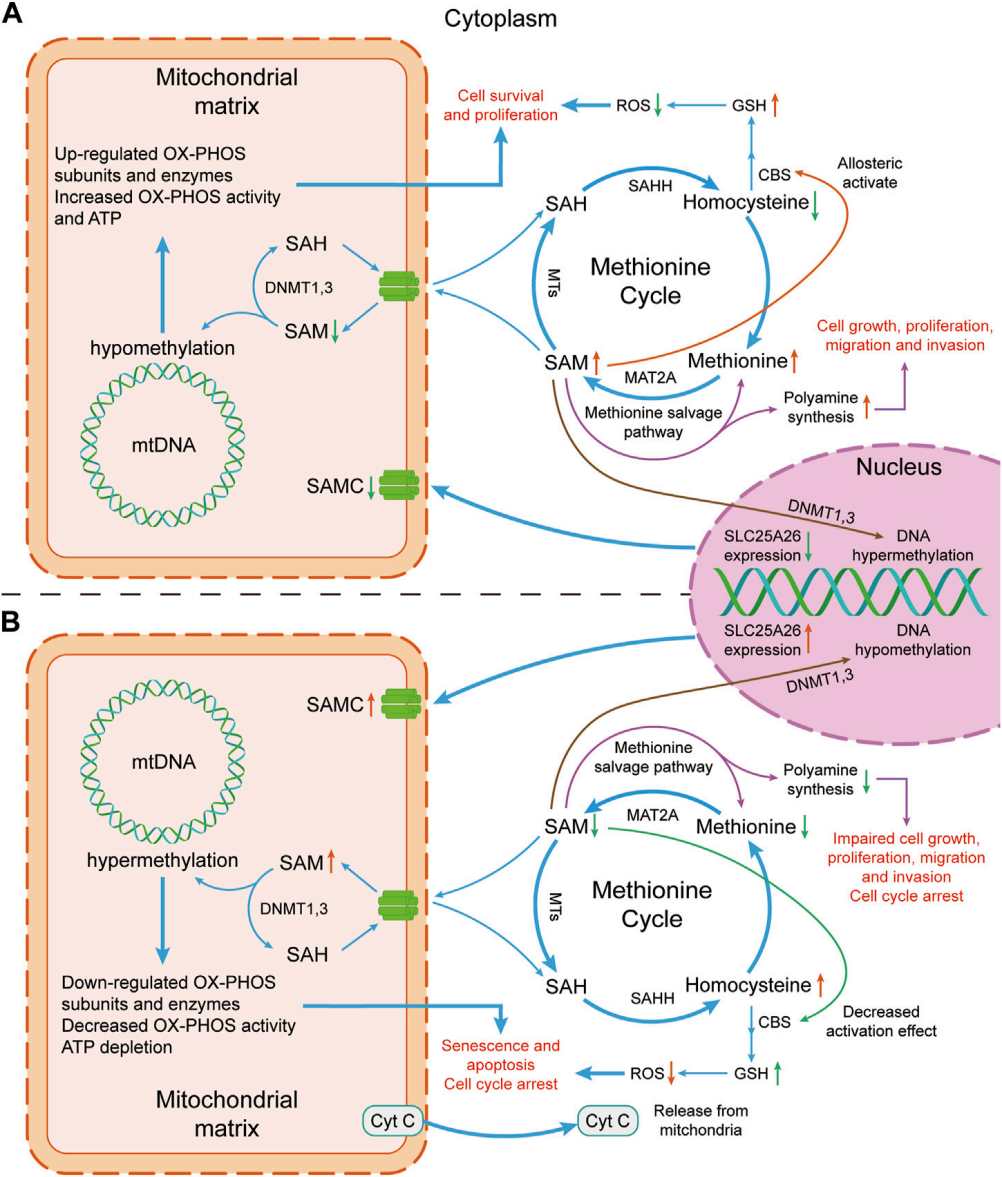
Mechanism diagram of the effect of altered *SLC25A26* expression level on cancer cells. **(A)** Lower expression level of *SLC25A26* may be beneficial to cancer cells. Lower expression level of *SLC25A26* reduces mitochondrial SAMC levels, thereby reducing mitochondrial uptake of cytosolic SAM, resulting in a decrease in mitochondrial SAM concentration and an increase in cytosolic SAM concentration. Such changes in SAM level will eventually lead to the growth, survival, proliferation, migration and invasion of cancer cells by promoting GSH synthesis and polyamine synthesis, as well as up-regulating mitochondrial respiratory enzyme levels. **(B)** Higher expression levels of *SLC25A26* may be detrimental to cancer cells. In contrast to panel **(A)**, higher *SLC25A26* expression level increases mitochondrial SAMC levels and therefore increases mitochondrial uptake of cytosolic SAM, resulting in an increase in mitochondrial SAM concentration and a decrease in cytosolic SAM concentration. Such changes in SAM level will eventually lead to senescence, apoptosis, cell cycle arrest of cancer cells, and inhibition of its growth, proliferation, migration and invasion by reducing GSH synthesis and polyamine synthesis, as well as down-regulating mitochondrial respiratory enzymes. SAH, S-adenosylhomocysteine; SAHH, S-adenosyl-L-homocysteine hydrolase; SAM, S-adenosylmethionine; SAMC, S-adenosylmethionine carrier; ROS, reactive oxygen species; GSH, glutathione; CBS, cystathione-β synthase; mtDNA, mitochondrial DNA; MTs, Methyltransferases; MAT2A, methionine adenosyltransferase 2A; DNMT, DNA methyltransferase; OX-PHOS, oxidative phosphorylation; Cyt C, cytochrome c.

Studies from clinical trials, and animal and cell models indicate that *SLC25A26* abnormal expression can regulate the occurrence and development of cancers. Its potential mechanisms are as follows (Figure 2).

### Affecting the Activity of Methionine Cycle Metabolism

The methionine cycle consists of a series of reactions that break down and regenerate methionine which is essential for many cellular functions, such as DNA and polyamine synthesis, histone and DNA methylation, and redox balance. Reports showed that the proliferation process of cancer cells is highly dependent on the methionine cycle [44]. Moreover, lung tumor-initiating cells showed highly elevated methionine cycle activity and transmethylation rates driven by MAT2A. High methionine cycling activity results in methionine consumption far exceeding its regeneration, leading to extrinsic methionine addiction. When the methionine cycle was, even transiently, inhibited by drug, the tumor-initiating ability of tumor-initiating cells was sufficiently decreased. At the same time, methionine cycling flux specifically affects the epigenetic state of cancer cells and drives cancer initiation [20]. Therefore, interference with methionine cycle is one of the targets of tumor therapy.

The novel copper complex CTB, a candidate anti-tumor compound, significantly inhibits the methionine cycle and promotes hepatocellular carcinoma cell senescence via inducing the accumulation of SAMC. siSLC25A26 treatment has opposite effects [28]. Therefore, the occurrence and development of cancers can be controlled by regulating the expression of *SLC25A26* to affect the activity of the methionine cycle metabolism.

### Regulating DNA Methylation

Cancer is an outcome of abnormal genetic and epigenetic changes. Epigenetic mechanisms, responsible for regulating gene expression without altering the DNA sequence, mainly include DNA methylation, histone modification, and chromatin remodelling. DNA methylation is the most studied mechanism [45]. The process of DNA methylation refers to the enzymatic addition of methyl groups to the cytosine residues by DNA methyltransferases, which relies on the availability of methyl groups and the function of the corresponding methyl donors and acceptors [11]. DNA methylation is a normal mechanism by which cells regulate gene expression in the mammalian genome. However, in 1983, Feinberg and Vogelstein first reported that there was extensive global loss of 5′-methylcytosine (the main form of DNA methylation) in colon cancers compared with the normal colon [46]. Subsequent studies confirmed that aberrant DNA methylation is a nearly universal finding in cancers and a major early contributor to cancer development [47, 48]. Therefore, regulating DNA methylation is one of the therapeutic targets for cancers.

No methylated sites were found in a CpG island in non-tumoral *SLC25A26* promoter, while in CaSki and HeLa cancer cells, 13 and 15 methylated sites of DNA were found, respectively. In CaSki cells (a cervical cancer cell), *SLC25A26* gene promoter hypermethylation results in downregulation of *SLC25A26* expression, which decreased SAM levels in mitochondria, induced hypomethylation of mitochondrial DNA, enhanced expression of key respiratory complex subunits, generation of mitochondrial ATP. At the same time, increased SAM in cytoplasm enhanced the methionine cycle, which reduces homocysteine and ROS but increases glutathione (GSH) that enhances cellular antioxidant defenses. All these events occur almost simultaneously to promote cancer cell survival and proliferation. On the contrary, overexpression of *SLC25A26* increased mitochondrial SAM level and enhanced hypermethylation of mitochondrial DNA, which inhibited expression of key respiratory complex subunits, and generation of mitochondrial ATP but enhanced release of cytochrome C (a trigger of type II apoptosis when released from the mitochondria). At the same time, methionine cycle was disrupted because SAM was in large quantities transported into mitochondria, which increases homocysteine and ROS and reduces GSH that reduces cellular antioxidant defenses. All these events occur almost simultaneously to arrest the cell cycle in the S phase and enhance cisplatin chemosensitivity [26].

Subsequently, Cianciulli et al. found that FOXD3, a member of the forkhead protein family, was a repressor of *SLC25A26* gene expression. Moreover, after CaSki cells were treated with folate, the repressive role of FOXD3 was completely abrogated via affecting methylation of FOXD3 gene promoter [25]. Additionally, studies on CTB have shown that the suppression of extra-mitochondrial methylation by CTB-induced *SLC25A26* overexpression can inhibit cancer cell genesis, progression, and proliferation by inhibiting the methylation of the telomerase reverse transcriptase promoter [28]. These studies showed that *SLC25A26* may regulate the occurrence and development of cancers via affecting DNA methylation.

### Regulating Cell Senescence

Cellular senescence, a stable cell cycle arrest that limits cell proliferation, is pivotal in various stages of tumorigenesis, including initiation, formation, and escape [49–51]. This process can also inhibit cancer development through autocrine and paracrine manners [49–52]. Consequently, therapies aimed at selectively enhancing senescence in cancer cells, known as “prosenescence” therapies, can be instrumental in anti-cancer treatment strategies [51].

In the human clinical liver cancer tissues with high expression of *SLC25A26*, proliferation index Ki67 had low expression and the senescence markers p16, p21, and HMGA1 had high expression, and the tissues with low expression of *SLC25A26* had opposite effects [28]. Moreover, during novel copper complex CTB-induced hepatocellular carcinoma cell senescence, the accumulation of SAMC is essential [28]. These results indicated that *SLC25A26* may regulate cancer cell senescence.

### Regulating Apoptosis

Apoptosis is considered to be the most vital form of cell death, which plays a crucial role for all multicellular organisms to regulate cell proliferation and maintain tissue homeostasis as well as get rid of unnecessary or harmful cells from an organism [53, 54]. Defects of apoptotic physiological mechanisms may lead to different human diseases, such as cancer. Cancer cells can alter apoptotic pathways through transcription, translation, and post-translation as well as escape apoptosis via several different strategies. Therefore, apoptosis has become one of the main molecular targets for drug discovery and development, especially for diseases such as cancer [55–57]. In CaSki cells, overexpression of *SLC25A26* promotes apoptosis, inhibits cell growth, and enhances chemosensitivity to cisplatin by reducing cytoplasmic SAM levels and increasing mitochondrial SAM, thereby suppressing methionine metabolism and methylation [26]. Moreover, several studies have demonstrated that restricting exogenous methionine intake in cancer cells can enhance control over various cancers and improve interventions with radiotherapy and chemotherapy for cancer [58–61]. Therefore, upregulating *SLC25A26* and limiting methionine intake in cancer cells may be able to further affect cellular methionine metabolism, potentially enhancing apoptosis and enhancing the effect of chemotherapy drugs.

### Regulating Immunity

Cancer, a genomic disease, prompts the immune system to recognize tumor antigens as foreign and triggers cellular immune responses [62, 63], inducing immune cells to infiltrate into the tumor microenvironment and regulate tumor progression [64–66]. Therefore, immunotherapy provides a more effective and less toxic alternative to chemotherapy for cancer patients [66–68], which is revolutionizing cancer treatment.

In patients with non-small cell lung cancer, immune infiltration analysis showed that *SLC25A26* expression was negatively correlated with 10-year survival [31]. SAM and histone methylation were supported by one-carbon metabolism to drive inflammatory macrophage infiltration [21]. Immuno-infiltration analysis also showed that *SLC25A26* expression in several tumors has a positive or negative correlation with the abundance of immune cell subsets around the tumor tissue [32]. Expression of *SLC25A26* in lower-grade glioma (GBMLGG) and glioblastoma (GBM), melanoma (SKCM), uveal melanoma (UVM), and colon and rectal cancer (COADREAD) has a significant negative correlation with the abundance of dendritic cells, M1 macrophages, and T helper 2 cells around them. On the contrary, expression of *SLC25A26* in cholangiocarcinoma (CHOL), esophageal cancer (ESCA), and sarcoma (SARC) has a significant positive correlation with the abundance of dendritic cells and M1 macrophages around them [32]. Moreover, since DC, M1 macrophages, and Th2 have different mechanisms to directly or indirectly inhibit or kill cancer cells, increased *SLC25A26* expression may promote the development of GBMLGG, SKCM, UVM, and COADREAD, whereas decreased *SLC25A26* expression is beneficial to inhibit or kill them. In contrast, low expression of *SLC25A26* may promote the development of CHOL, ESCA, and SARC, while high expression of *SLC25A26* is beneficial to inhibit or kill them [32].

These results indicated that *SLC25A26* may regulate cancer cell development via affecting the immune system, which may be a research hotspot of tumor treatment in the future and provide a new direction for tumor immunotherapy.

## Potential Role of *SLC25A26* in Cancer Prognosis

The low expression levels of eight genes located on chromosome 3p12-14, including *SLC25A26*, have been verified to be associated with poor prognosis in patients with cervical squamous cell carcinomas (SCC). In addition, SCC is more likely to have chromosome 3p12-14 loss and is associated with aggressive tumour growth compared to precancerous lesions. Therefore, these eight genes may contribute to evaluating cancer aggressiveness in the early invasive stage. Based on the low intratumor heterogeneity of these genes, the gene expression level can be obtained by FISH for clinical diagnostic testing [43].

*SLC25A26* expression is detected as low in HCC tissues, which is correlated with a high level of proliferation index Ki67 and low expression of senescence markers p16, p21 and HMGA1 (28). Therefore, we speculate that the low expression of *SLC25A26* in HCC tissues may be related to the poor prognosis of patients.

For NSCLC, a total of 5 genes including *SLC25A26* were selected and made as a risk assessment model. In the model, high expression of *SLC25A26* in NSCLC patients is positively correlated with the risk score and predicts a poor 10-year survival rate [31].

In brief, these studies suggest that *SLC25A26* expression level may be differently correlated with the prognosis in different cancers. However, the relationship between *SLC25A26* expression and the prognosis of other cancers as well as the specific predictive indicators remain to be investigated. In addition, it should be explored whether *SLC25A26* expression is relevant for predicting, detecting, or diagnosing certain cancers.

## *SLC25A26* Mutation and Mitochondrial Diseases

It has also been reported that *SLC25A26* mutation may lead to mitochondrial defects and then induce a mitochondrial disease called combined oxidative phosphorylation deficiency 28 (COXPD28).

COXPD28 is an autosomal recessive genetic disease first identified by Kishita et al. in 2015 in three unrelated severe infant cases [69]. Subsequently, two new infants with COXPD28 were reported in 2022 [70, 71]. The five patients presented with varying degrees of clinical symptoms, ranging from mild muscle weakness, cardiopulmonary insufficiency, and developmental delay to respiratory and circulatory/multiple organ failure and death, accompanied by hyperlactatemia. Muscle biopsy revealed varying degrees of decrease in mitochondrial complexes I, III, and IV [69]. Further research on infants with COXPD28 indicated that the *SLC25A26* mutation severely impairs the function of SAMC in transporting SAM into the mitochondria [69], which may lead to a more severe early-onset phenotype. This mutation results in a deficiency of mitochondrial SAM input, leading to a lack of methylation substrates and impaired mitochondrial biosynthesis. Specifically, mtRNA methylation is impaired, affecting rRNA stability and tRNA maturation, further impacting ribosomal assembly and *de novo* translation processes in mitochondria. The steady-state levels of COXII, a subunit of complex IV, was reduced, and the methylation of ADP/ATP translocase genes ANT1 and ANT2, as well as the electron transfer flavoprotein ETFβ, were found to be hypomethylated. The biosynthesis of LA [72], which relies on SAM for methylation, is impaired, leading to reduced levels of the pyruvate dehydrogenase complex E2 (PDHC-E2) and the α-ketoglutarate dehydrogenase E2 (α-KGDH-E2), of which LA is a subunit, and further affecting the activity of PDH and α-KDGH. The biosynthesis of CoQ10, an electron carrier in the mitochondrial oxidative respiratory chain, is also impaired, leading to a significant decrease in mitochondrial ATP production [69].

Additionally, three adult female patients with milder phenotypes of COXPD28 have been reported [73, 74]. The common symptoms of these three patients were muscle weakness, gastrointestinal discomfort, and hyperlactacidemia. Independent symptoms include respiratory and multi-organ failure, exercise intolerance with muscle pain and disability. Studies in adult cases of COXPD28 suggest that the *SLC25A26* mutation primarily affects the function of SAMC in transporting SAH into the cytoplasm, which may result in a milder late-onset phenotype. In mouse embryonic fibroblast models, cells with the *SLC25A26* mutation also showed reduced mitochondrial LA levels and PDH activity [73]. In *Drosophila Melanogaster* larvae with the *SLC25A26* mutation, a decrease in the pentose phosphate pathway and bile acid synthesis was found, while iron-sulfur clusters and ubiquinone biosynthesis proteins were upregulated, and OXPHOS components UQCR-11 and COX7A proteins both showed increased expression, along with biomarkers of mitochondrial methylation defects [21], including the absence of glycine-N-methyltransferase (GNMT) and an increase in serine biosynthetic enzyme phosphoserine phosphatase (PSPH) [73].

In conclusion, *SLC25A26* mutations impair mitochondrial RNA stability, protein synthesis, LA, and CoQ10 biosynthesis, which further affect TCA and the mitochondrial oxidative respiratory chain, resulting in the intractable mitochondrial disease COXPD28. This also emphasizes the importance of *SLC25A26* for mitochondrial function. By the way, in patients with major depression disorder, the plasma SAMC level was found to be significantly increased [75].

## Conclusion and Perspective

In conclusion, we briefly reviewed the essential roles of SAMC and its encoding gene, further empathizes association of *SLC25A26* aberrant expression and some cancers, as well as potential mechanisms for *SLC25A26* regulating the occurrence and development of cancers including regulating the activity of methionine cycle metabolism, DNA methylation, senescence, apoptosis and immune cell infiltration. Next, we summarized its potential in cancer prognosis and finally emphasized its importance in mitochondria function by reviewing an intractable mitochondrial disease named COXPD28 caused by *SLC25A26* mutation.

However, the role of *SLC25A26* in cancer regulation remains to be fully elucidated. Key questions include how varying expression levels of *SLC25A26* affect the methylation of genes in the nucleus or mitochondria, and how these changes regulate various aspects of cancer through specific pathways. Additionally, the impact of *SLC25A26* expression on the infiltration of immune cell subsets adjacent to different cancer cells and the abundance of cancer cells, and its subsequent influence on cancer progression through immune modulation, is an area that needs further investigation. The potential of *SLC25A26* as a target for cancer therapy is yet to be fully exploited, either. For instance, the discovery of potent inhibitors of the expression product of *SLC25A26*, SAMC, has been made [9], but their effects on cancer have not yet been studied. Moreover, the downregulation of *SLC25A26* in invasive cervical SCC may enhance invasiveness and resistance to treatment [43]. This finding prompts a deeper exploration into the mechanisms linking *SLC25A26* downregulation with cancer cell invasiveness and treatment resistance, potentially revealing new strategies to reduce cancer lethality and improve therapeutic efficacy. Furthermore, the novel copper complex CTB has been shown to inhibit tumorigenesis *in vivo* by up-regulating *SLC25A26* expression [28], while MC38 cells with *SLC25A26* knockout exhibited massive cell death [32], and *SLC25A26* knockout mice were embryonically lethal [21]. These observations suggest that both extremely high and low levels of *SLC25A26* expression could lead to the inhibition or death of cancer cells via distinct pathways, indicating that *SLC25A26* inhibitors or activators may represent novel cancer gene therapies awaiting further research. Besides, further investigation is needed to determine the role of *SLC25A26* in predicting, diagnosing, and prognosing cancer.

Overall, *SLC25A26* may regulate the occurrence and development of cancers via many pathways, which suggests that *SLC25A26* will be a potential new therapeutic target for cancers.
